# Identifying neurological comorbidities in obstructive sleep apnea patients through polysomnography

**DOI:** 10.1007/s11325-020-02231-w

**Published:** 2020-10-22

**Authors:** Lothar Burghaus, Lisa Piano, Gereon R. Fink, Lennart Knaack

**Affiliations:** 1 Department of Neurology, Heilig Geist-Hospital, Grasegger Str. 105, 50737 Cologne, Germany; 2 Department of Neurology, University Hospital Cologne, Cologne, Germany; 3 Intersom Köln, Center of Sleep Medicine and Sleep Research, Cologne, Germany; 4 Inst. of Neuroscience and Medicine (INM-3), Forschungszentrum Juelich, Juelich, Germany

To the Editor,

Obstructive sleep apnea (OSA) is a common disease characterized by recurrent respiratory flow limitations and closure of the upper airway accompanied by repetitive hypoxia during sleep. Increased arousal activity, sleep fragmentation, and disturbed sleep architecture cause cardinal symptoms like excessive daytime sleepiness and impaired quality of life. OSA is associated with an increased risk of complications such as stroke or cardiovascular events. In males with severe OSA, the risk of a cerebrovascular event is nearly three times higher [1]. Likewise, sleep-related breathing and sleep-wake disturbances frequently occur in ischemic stroke. More than 50% of stroke patients have sleep-related breathing disturbances, presenting with obstructive, central, or mixed apneas [2]. Recently, in this journal, Leino et al. discussed specific polysomnographic features of acute stroke and TIA patients with OSA [3].

Stroke patients are of particular interest because OSA is a significant risk factor for cerebral ischemia, and, vice versa, cerebrovascular lesions can cause sleep disorders [4]. In stroke patients, the treatment of OSA impacts the outcome. Stroke patients with an apnea-hypopnea index (AHI) > 20 effectively treated by continuous positive airway pressure (CPAP) had a significantly better neurological outcome after 1 month than the control group without CPAP treatment [5]. Consequently, stroke patients should be screened for sleep apnea after acute cerebral ischemia to start treatment as soon as possible [4].

We screened a large group of sleep laboratory patients with OSA for neurological comorbidities and looked for abnormalities of polysomnographic parameters that might help to identify neurological comorbidities.

We analyzed 776 patients diagnosed with at least mild OSA (AHI ≥ 5/h). According to current standards, all patients had a suspected diagnosis of OSA and, therefore, underwent polysomnography. We next compared polysomnographic parameters between patients with OSA who additionally had a history of neurological disease and those who did not have any diagnosed neurological comorbidities. The data were also analyzed for each neurological disease, e.g., cerebral ischemia or neurodegenerative diseases. In a further step, significant differences in the parameters were examined as to whether or not they detected yet undiagnosed neurological comorbidity.

Cerebral ischemia as a comorbidity was found in 27 of the 776 patients (mean age 68.7 years ± 10.7 versus 56.9 years ± 12.4). These patients showed particularly poor sleep efficiency and a higher proportion of apnea in the AHI (47.5% versus 28.7%). Mean oxygen desaturation index (ODI) was 29.2 (± 18.1) and oxygen saturation was lower than 90% in 9.4% (± 14.0) of measuring time. Further information on the patients is listed in Table 1.

**Table 1:** Polysomnographic indices in patients with and without cerebral ischemia

	Patients with cerebral ischemia (*n* = 27)		Control group (*n* = 749)	
	M	SD	M	SD
Age (years)	68.67	10.69	56.87	12.35
Neck circumference (cm)	42.15	2.60	41.66	3.85
BMI (kg/m^2^)	27.93	2.83	29.54	5.05
AHI	30.33	19.74	30.80	21.63
AI	17.67	18.95	10.72	13.47
HI*	12.67	8.62	20.07	14.84
AI P*	47.50	30.92	28.73	23.49
HI P*	52.50	30.92	71.27	23.49
RDI	34.36	18.31	35.63	20.80
ESS*	6.26	3.16	7.65	4.67
O_2_	94.60	1.99	94.28	1.68
O_2_ min	82.52	6.94	82.52	7.16
O_2_ mean	6.12	1.91	5.88	1.97
PLMI	27.20	25.89	18.98	23.71
REM%	12.60	5.63	14.85	6.73
NREM1%	23.38	17.31	19.65	17.51
NREM2%	46.99	18.83	47.87	15.24
NREM3%	17.04	11.82	17.63	10.04
REM latency	145.07	64.71	13.01	82.26
Sleep efficiency*	74.35	14.69	81.16	11.57
Sleep latency	34.75	49.13	20.43	24.50
Arousal index	45.95	20.30	44.84	19.66
SWS-Latency*	118.67	120.14	77.92	104.50
Snore index	27.01	21.65	24.50	20.11

*BMI* body mass index, *AHI* apnea + hypopnea index, *AI* apnea index, *HI* hypopnoea index, *AI-P* apnoea percentage of total AHI, *HI-P* hypopnoea percentage of total AHI, *RDI* respiratory disturbance index, *ESS* Epworth Sleepiness Scale, *O*_*2*_ oxygen saturation (min: lowest value of oxygen saturation, mean: mean value of oxygen desaturation), *PLMI* periodic limb movement index, *REM* rapid eye movement sleep, *SWS* slow wave sleep, *M* mean value, *SD* standard deviation

*p ≤ 0.05

**Fig. 1:**
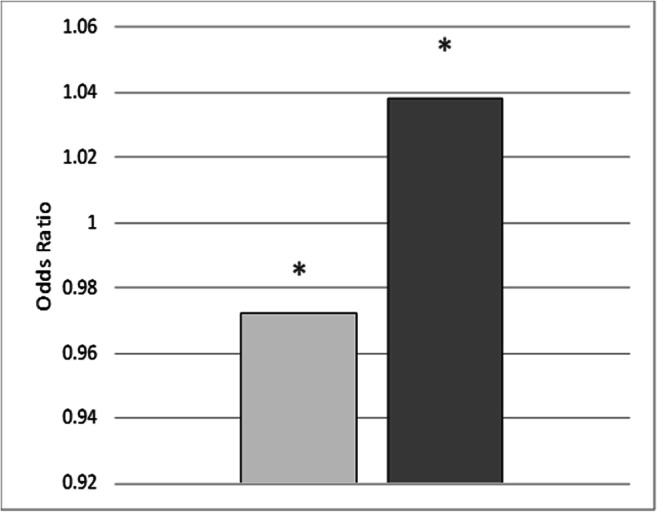
Logistic regression; independent variables: relative proportion of apnea in total AHI (left column) and sleep efficiency (right column), dependent variable: cerebral ischemia; * *p* ≤ 0.05

The key question is , conversely, if patients who suffer from a particularly high proportion of apnea in their AHI and poor sleep efficiency may be those patients that have already suffered from cerebral ischemia. This question should be answered using logistic regression and calculating the odds ratio. The regression coefficient showed (*p* = 0.004) that the lower the relative apnea index based on the total AHI (OR = .972) and the higher the sleep efficiency (OR = 1.038), the more likely the patient was not in the group with cerebral ischemia (Fig. 1).

Patients with neurological disorders such as stroke or neurodegenerative diseases should be screened for sleep-related breathing disorders regularly. In this study, we demonstrated that vice versa, regular polysomnographic parameters may be indicative of a cerebrovascular disease in patients with OSA. Therefore, we suggest that patients with OSA who present with a high relative apnea index and poor sleep efficiency in standardized polysomnography should be screened for cerebrovascular diseases. To what extent patients with OSA who have polysomnographic findings may benefit from a neurological screening for previously unknown cerebrovascular diseases is the subject of our further research.
